# Retrospective evaluation of natural course in mild cases of *Mycobacterium avium* complex pulmonary disease

**DOI:** 10.1371/journal.pone.0216034

**Published:** 2019-04-25

**Authors:** Yoshifumi Kimizuka, Yoshihiko Hoshino, Tomoyasu Nishimura, Takahiro Asami, Yumi Sakakibara, Kozo Morimoto, Shinji Maeda, Noboru Nakata, Takayuki Abe, Shunsuke Uno, Ho Namkoong, Hiroshi Fujiwara, Yohei Funatsu, Kazuma Yagi, Toshihide Fujie, Makoto Ishii, Naohiko Inase, Satoshi Iwata, Atsuyuki Kurashima, Tomoko Betsuyaku, Naoki Hasegawa, for the Non-Tuberculous Mycobacteriosis-Japan Research Consortium (NTM-JRC)

**Affiliations:** 1 Division of Pulmonary Medicine, Department of Medicine, School of Medicine, Keio University, Shinjuku, Tokyo, Japan; 2 Division of Infectious Diseases and Respiratory Medicine, Department of Internal Medicine, National Defense Medical College, Tokorozawa, Saitama, Japan; 3 Leprosy Research Center, National Institute of Infectious Diseases, Higashimurayama, Tokyo, Japan; 4 Health Center, Keio University, Shinjuku, Tokyo, Japan; 5 Department of Integrated Pulmonology, Tokyo Medical and Dental University, Bunkyo, Tokyo, Japan; 6 Respiratory Disease Center, Fukujuji Hospital, Anti-tuberculosis Association, Kiyose, Tokyo, Japan; 7 Faculty of Pharmaceutical Sciences, Hokkaido University of Science, Sapporo, Hokkaido, Japan; 8 Department of Preventive Medicine and Public Health, Biostatistics Unit at Clinical and Translational Research Center, School of Medicine, Keio University, Shinjuku, Tokyo, Japan; 9 Center for Infectious Diseases and Infection Control, School of Medicine, Keio University, Shinjuku, Tokyo, Japan; 10 Department of Infectious Diseases, Keio University School of Medicine, Shinjuku, Tokyo, Japan; Toranomon Hospital, JAPAN

## Abstract

**Background:**

There is no proven management for mild cases of *Mycobacterium avium* complex (MAC) pulmonary disease, who do not immediately receive treatment and are managed with observation alone, because its long term-natural course, factors predictive of deterioration, and the effect of treating the disease remain unclear. Thus, we sought to investigate the natural course of mild cases of MAC pulmonary disease.

**Methods:**

We conducted a multicenter retrospective study. Sixty-five patients with mild MAC pulmonary disease in whom treatment was withheld for at least 6 months after diagnosis were retrospectively recruited after a review of 747 medical records. Longitudinal changes in clinical features were evaluated by using a mixed effects model.

**Results:**

Mean follow-up was 6.9 ± 5.7 years. During the follow-up period, 15 patients (23%) required treatment and 50 (77%) were managed with observation alone. At diagnosis, 65 patients had nodular bronchiectatic disease without fibrocavitary lesions. Among clinical features, mean body mass index (BMI), forced expiratory volume in 1 second as percent of forced vital capacity (%FEV_1_), nodular lung lesions, and bronchiectasis worsened significantly in the observation group during follow-up. In the treatment group, BMI, and %FEV_1_ were stable, but bronchiectasis significantly worsened. At diagnosis, the polyclonal MAC infection rate in the treatment group was higher than that in the observation group. Other microbiological factors, such as insertion sequences, did not differ significantly between the groups.

**Conclusions:**

Mild MAC pulmonary disease progresses slowly but substantially without treatment. Treatment prevents the deterioration of the disease but not the progression of bronchiectasis. Polyclonal MAC infection is a predictor of disease progression.

## Introduction

The prevalence of nontuberculous mycobacterial (NTM) pulmonary disease is increasing around the world, especially in developed countries [1–8]. Although substantial geographic differences are seen in the distribution of the pathogens responsible for NTM pulmonary disease, the *Mycobacterium avium* complex (MAC) is the most common pathogen in Japan and in other developed, pan-Pacific countries [1–9]. Radiographic features of MAC pulmonary disease (pMAC) are classified into two main types, fibrocavitary and nodular bronchiectatic. A large proportion of patients with the nodular bronchiectatic form of pMAC are middle-aged or elderly female patients without pulmonary comorbidities or history of smoking [10].

The official statement of the American Thoracic Society (ATS) and Infectious Diseases Society of America (IDSA) on the management of NTM diseases suggests that pMAC treatment should only be initiated when the symptoms or radiologic findings worsen [10]. Therefore, patients with mild pMAC, such as nodular-bronchiectatic disease, do not immediately receive treatment and are managed by observation alone [9, 10]. However, there is no clear-cut management strategy for mild pMAC because its long-term natural course, factors predicting its deterioration, and the effects of treatment are still unclear.

Variable-number tandem-repeat (VNTR) analysis has recently been used to determine MAC strain genotypes to ascertain whether infection is polyclonal or monoclonal, even though the precise relationship between polyclonality and pathogenicity also remains unclear. Nonetheless, infection was found to be largely monoclonal in fibrocavitary disease but polyclonal in nodular-bronchiectatic disease [11], and some studies have shown an association between the prevalence of specific plasmids and the pathogenesis of MAC infection [12–14]. These observations suggest that bacterial factors may be related to the pathogenesis of pMAC, but bacterial factors affecting mild pMAC have not yet been investigated.

Therefore, we evaluated both clinical and bacterial characteristics in patients with mild pMAC in whom treatment had been initially withheld. To determine if clinical and bacterial features can predict deterioration of mild pMAC, we compared these clinical and bacterial features between patients who were managed by observation alone and those who eventually required treatment.

## Methods

### Ethical approval

The study protocol was reviewed and approved by all three institutional ethics committees (Keio University School of Medicine and Hospital, No. 20080131; Tokyo Medical and Dental University, No. 1589; and Fukujuji Hospital; Title: ‘Clinical, radiological, bacteriological research in MAC pulmonary disease'). This protocol was also approved by the medical research ethics committee of the National Institute of Infectious Diseases in Japan for use on human subjects (No. 408).

### Patients

**Fig 1:**
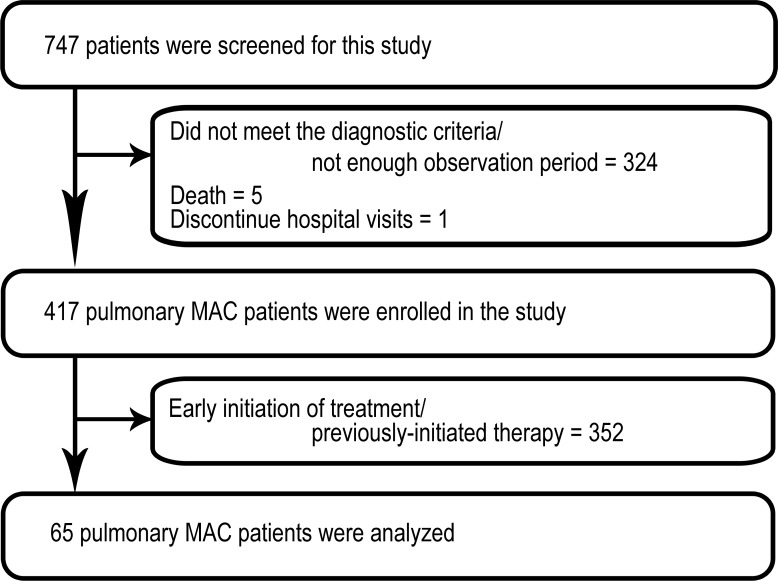
Flow chart of the study of patients with mild *Mycobacterium avium* complex pulmonary disease. MAC = *Mycobacterium avium* complex.

We retrospectively reviewed the medical records of 747 patients, either diagnosed with or suspected of pMAC, evaluated in individual centre of three tertiary hospitals in Tokyo, Japan (The research periods: Keio University Hospital between January 2001 and July 2013, Tokyo Medical and Dental University Hospital between January 2007 and July 2013, and Fukujuji Hospital between September 2012 and July 2013). We identified 65 cases of pMAC on the basis of the following inclusion criteria: (a) satisfied the diagnostic criteria of ATS and IDSA [10], (b) had no history of pulmonary comorbidities or administration of antibiotics for more than two weeks and (c) treatment had been withheld for at least 6 months after diagnosis (Fig 1). All patient data were fully anonymized before we accessed.

### Study design

This was a retrospective study. After an inclusion period of at least 6 months without treatment after diagnosis, patients were categorised as either treated (treatment group) or untreated (observation group; S1 Fig), on the basis of whether or not treatment (triple drugs regimen of clarithromycin, rifampicin, and ethambutol) was initiated by their primary physicians within the observation period. In accordance with the Japanese Society for Tuberculosis and ATS/IDSA guidelines, treatment has been carried out for at least 12 months after sputum conversion. The observation group comprised patients in whom treatment had been withheld throughout the observation period. Clinical features including patient characteristics, body mass index (BMI) and pulmonary function test, bacteriologic characteristics, and chest radiographic findings were compared between the two groups.

### Evaluation of clinical characteristics

At the first visit, patients were interviewed about characteristics and past medical history. Patients visited each hospital every three to six months. At every visit, primary physicians assessed patients for symptoms such as sputum, cough and fever, and chest X-rays (CXRs) were performed. When the patients were judged to require chest computed tomography (CT) by their primary physician, CTs were performed. When patients produced sputum, sputum cultures were performed. The initiation of treatment was decided by each primary physician based on the deterioration of clinical features.

### Evaluation of bacterial factors

MAC was isolated from the sputum or bronchial washings obtained from patients at diagnosis and as described previously [15, 16]. For VNTR analysis, positive cultures from patients’ samples at diagnosis were subcultured using the Bactec MGIT 960 Mycobacterial Detection System for a maximum of 6 weeks. Identification of insertion sequences (IS1245, IS1311, IS*Mav6*, or IS*Mav6* inserted into the upstream region of the culture filtration protein 29 gene [IS*Mav6* in *cfp*29]) and plasmid sequences (pMAH135, pLR7, pVT2, pMAH135+ pVT2, or pMAH135 + pLR7 + pVT2) were performed as previously described (S1 Table) [13, 14, 17].

VNTR analysis was performed as described previously [18, 19]. Sequences of the primers used for amplification of 11 *Mycobacterium avium* tandem repeat (MATR) loci (MATR-1, 2, 3, 6, 7, 8, 9, 11, 13, 14 and 16) and 5 Higashi Nagoya tandem repeat (HNTR) loci (HNTR-1, 2, 3, 5 and 6) were selected. VNTR typing was performed using the Ex Taq with the GC PCR buffer I (Takara Bio). The microchip electrophoresis analysis system (MCE-202 MultiNA; Shimadzu Corporation, Kyoto) was used to determine the sizes of the amplified DNA fragments, calibrate VNTR analysis and analyse 16 MATR loci, 5 HNTR loci (to evaluate *M*. *avium* in sample) [18, 19], and 16 VNTR loci (to evaluate *Mycobacterium intracellulare* in sample) [20]. Respective copy numbers were calculated from the size and assigned according to the number of repeats for each locus. The copy number of *M*. *avium* strain 104 was used to define each locus in the MAC and that of strain ATCC13950 was used to define each locus in *M*. *intracellulare*. Polyclonal infection with MAC was defined on the basis of VNTR analysis as showing a difference at two or more loci in *M*. *avium* or *M*. *intracellulare*. Mixed infection was defined as the isolation of additional mycobacterial species from a sample. The dissimilarity among mycobacterial genotypes was evaluated by using the Manhattan distance and the minimum spanning tree (MST). MSTs of *M*. *avium* isolates based on 16 VNTR loci were drawn using the BioNumerics program (Applied Maths NV, Sint-Martens-Latem, Belgium) [21].

### Radiographic findings

The interpretation of CXRs and CTs was supervised by a board-certificated radiologist, and seven board-certificated pulmonologists with over 10 years of clinical experience reviewed the CXRs using the scoring system (S2 Fig) [22]. Cases were randomly allocated to reviewers, and each case was scored by two reviewers who were blinded to relevant clinical information. Reviewers assessed four types of pulmonary lesions, namely N (nodules), I (infiltrate or consolidation), C (cavity), or E (ectasis), to calculate the radiologic findings score. Each lung field on the CXR and CT image was divided into three zones, and the total score for an entire lung was calculated for each type of lesion by adding the results from two reviewers. In addition, we evaluated the interobserver concordance of radiographic scores on CXR and CT (S2 Table and S3 Table).

### Statistical analysis

Data are presented as mean ± standard deviation (SD) for continuous variables. Differences between subjects for categorical variables were tested using the *χ*^2^ test or using a two-tailed, unpaired *t* test for comparing means of continuous data. Average longitudinal profiles were estimated on the basis of a mixed effects model that contained random intercepts for each patient and time as a factor that was adjusted for the variation in the length of follow-up for each patient. The association between clinical and radiologic findings and bacterial factors was assessed using a multiple linear regression model. Manhattan distances in VNTR analysis were calculated as described previously [17]. The significance level for all tests was set at 0.05 (two-sided). All statistical analyses were performed using a commercial software package (SPSS Statistics 22.0, IBM, Inc., Armonk, NY, USA).

## Results

### Patient characteristics

Of the 65 patients, 43 (66.2%) were female (Table 1), and none had an immunodeficiency syndrome or were being treated with immunosuppressive agents. The mean follow-up period after diagnosis of pMAC was 6.9 ± 5.7 yr.

During this follow-up period, 15 patients (23%) required treatment, and 50 patients (77%) were managed with observation alone; the overall duration of the follow-up period after diagnosis was not significantly different between the treatment and observation groups (7.5 ± 4.8 vs. 6.7 ± 5.9 yr, *P* = 0.679). In the treatment group, patients were observed for a mean period of 4.9 ± 4.8 yr before initiation of treatment. The reasons for initiating treatment were worsening of symptoms (*n* = 7 patients, of which 3 had haemoptysis, 3 had sputum and 1 had cough), increasing bacterial load in respiratory specimens (*n* = 6), worsening of radiographic findings on CXR (*n* = 5, four of 5 were also evaluated by CT) and others (*n* = 2). According to published classification criteria [23], all patients from both groups had nodular-bronchiectatic disease without fibrocavitary lesions. Although the radiographic scores at diagnosis on CT didn’t show the significant difference between two groups, the initial radiographic scores at diagnosis on CXR were significantly higher for nodules and ectasis (nodule: 6±3.2 vs 11±3.1; ectasis: 5±3.0 vs 9±3.4, respectively observation vs treatment group, both *P* < 0.001) in the treatment group than in the observation group.

**Table 1:** Characteristics of patients with mildly symptomatic *Mycobacterium avium* complex pulmonary disease*.

	Total n = 65	Untreated group n = 50	Treated group n = 15	*P* value†
Sex	Male 22	Male 21	Male 1	***P* = 0.011**‡
	Female 43	Female 29	Female 14	
Age (years)	65 ± 12.7	66 ± 13.5	62 ± 9.8	*P* = 0.348
Smoking status				
Current + former smoker	12 (18.5)	10 (20.0)	2 (16.7)	*P* = 0.559
(Brinkman index)	447.9 ± 334.6	410.0 ± 238.6	550.0 ± 226.3	*P* = 0.406
Non-smoker	53 (81.5)	40 (80.0)	13 (86.7)	
Drinking history				*P* = 0.245
Habitual drinker	14 (21.5)	13 (26.0)	1 (6.7)	
Occasional drinker	7 (10.8)	5 (10.0)	2 (13.3)	
Non-drinker	44 (67.7)	32 (64.0)	12 (80.0)	
Body mass index§	21.6 ± 2.5	21.7 ± 2.6	20.8 ± 2.9	*P* = 0.863
%VC (%) ||	89.8 ± 16.0	88.5 ± 16.7	95.5 ± 12.7	*P* = 0.309
%FEV_1.0_ (%) ||	90.3 ± 26.6	91.7 ± 27.1	87.6 ± 25.8	*P* = 0.500
Peak Flow (l/sec) ||	6.09 ± 1.86	6.30 ± 1.91	5.30 ± 1.51	*P* = 0.176
V_50_ (l/sec) ||	2.19 ± 1.08	2.33 ± 1.08	1.68 ± 0.98	*P* = 0.133
V_25_ (l/sec) ||	0.61 ± 0.38	0.66 ± 0.38	0.44 ± 0.36	*P* = 0.161
Radiological findings score (CXR)				
Nodule	7 ± 3.9	6 ± 3.2	11 ± 3.1	***P* < 0.001**‡
Infiltrate	3 ± 3.9	3 ± 4.1	5 ± 2.7	*P* = 0.069
Cavity	0 ± 0.5	0 ± 0.5	0 ± 0.5	*P* = 0.926
Ectasis	6 ± 3.5	5 ± 3.0	9 ± 3.4	***P* < 0.001**‡
Radiological findings score (CT)**	(n = 60)	(n = 47)	(n = 13)	
Nodule	8 ± 4.3	8 ± 4.5	8 ± 3.7	*P* = 0.968
Infiltrate	3 ± 3.5	3 ± 3.7	4 ± 2.7	*P* = 0.527
Cavity	0 ± 0.5	0 ± 0.5	0 ± 0.6	*P* = 0.922
Ectasis	7 ± 4.2	7 ± 4.3	7 ± 4.1	*P* = 0.539

%VC, vital capacity as percent of predicted; %FEV_1_, forced expiratory volume in 1 s as percent of forced vital capacity; V50, flow at 50% of forced vital capacity; V25, flow at 25% of forced vital capacity.

*Data are presented as mean ± SD or n (%).

†: Differences between observation and treatment groups were tested using the *χ*^2^ test for categorical data and an unpaired *t* test for comparison of means.

‡: *P* < 0.05.

§:BMI at diagnosis are shown. *n* = 52, 39, 13.

||: Respiratory function test result are shown. *n* = 38, 30, 8.

**: Radiological findings score on CT are shown. *n* = 60, 47, 13.

### Clinical and radiographic characteristics of the observation group

**Fig 2:**
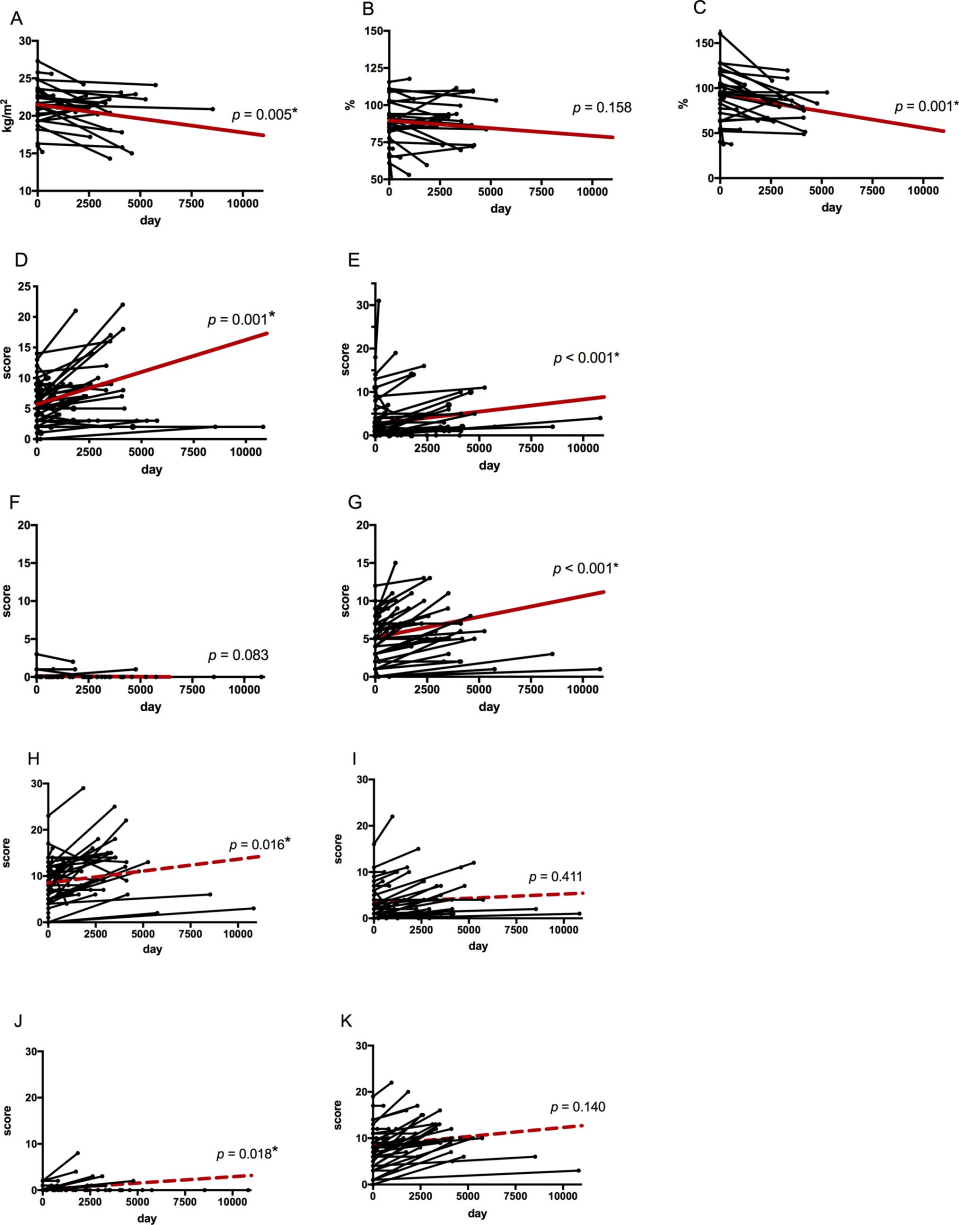
Longitudinal changes in clinical features of the untreated group. The horizontal axis shows the observation period. The vertical axis shows clinical features or radiographic scores of chest X-ray (CXR) and chest computed-tomography (CT). Each bold red line represents the average profile of the group using the mixed effect model. A: Body mass index (BMI); B: %VC (vital capacity as percent of predicted); C: %FEV_1_ (Forced expiratory volume in 1 second as percent of forced vital capacity); and radiologic scores of D: nodule (CXR), E: infiltrate (CXR), F: cavity (CXR), G: ectasis (CXR), H: nodule (CT), I: infiltrate (CT), J: cavity (CT), and K: ectasis (CT). *: Asterisks denote significant changes in the entire group through the observation period. Radiological findings score on CXR are shown. *n* = 50, 50. Radiological findings score on CT are shown. *n* = 47, 45.

In the observation group (Table 2), BMI and forced expiratory volume in 1 s as percent of forced vital capacity (%FEV_1_) were significantly lower at the end of follow-up than at diagnosis (BMI: 21.7 ± 2.6 vs 20.6 ± 2.7, *P* = 0.012; %FEV_1_: 91.7 ± 20.8 vs 79.8 ±20.8, *P* = 0.002, respectively at diagnosis vs last visit), whereas radiographic scores (nodules and ectasis) were significantly higher (CXR: nodules: 5.6 ± 3.2 vs 7.3 ± 5.0 *P* = 0.001; infiltrates: 3.0 ± 4.1 vs 4.6 ± 5.9, *P* < 0.001; ectasis: 4.9 ± 3.0 vs 6.5 ± 3.5, *P* < 0.001, respectively at diagnosis vs last visit, CT: nodules: 8.4 ± 4.5 vs 11.4 ± 5.4 *P* = 0.005; ectasis: 7.4 ± 4.3 vs 10.6 ± 4.0, *P* < 0.001, respectively at diagnosis vs last visit). These longitudinal changes in clinical and radiologic features were evaluated using the mixed effects model, while allowing for statistical correction of the effect of variable duration of observation for each patient (Fig 2). The model showed that BMI and %FEV_1_ significantly decreased and that the scores for nodules significantly increased (BMI: *β* = −0.00037, *P* = 0.005; %FEV_1_: *β* = −0.00368, *P* = 0.001; CXR: nodules: *β* = 0.000766, *P* = 0.001; CT: nodules: *β* = 0.000936, *P* = 0.016). In addition, the scores for ectasis of CXR and CT apparently increased, while those of CT did not show a significant difference. These results indicated that observation alone slightly but significantly deteriorates BMI, respiratory functions and radiological findings.

**Table 2:** Longitudinal changes in clinical features in the observation group.

	At diagnosis	Last visit	*P* value
Body mass index†	21.7 ± 2.6	20.6 ± 2.7	*P* = 0.012*
%VC ‡	88.5 ± 16.7	86.2 ± 17.4	*P* = 0.242
%FEV_1.0_ ‡	91.7 ± 20.8	79.8 ± 20.8	*P* = 0.002*
Radiographic score (CXR) §			
Nodule	5.6 ± 3.2	7.3 ± 5.0	*P* = 0.001*
Infiltrate	3.0 ± 4.1	4.6 ± 5.9	*P* < 0.001*
Cavity	0.1 ± 0.5	0.1 ± 0.4	*P* = 0.569
Ectasis	4.9 ± 3.0	6.5 ± 3.5	*P* < 0.001*
Radiographic score (CT) ||			
Nodule	8.4 ± 4.5	11.4 ± 5.4	*P* = 0.005*
Infiltrate	2.9 ± 3.7	4.6 ± 4.7	*P* = 0.065
Cavity	0.2 ± 0.5	0.6 ± 1.5	*P* = 0.050
Ectasis	7.4 ± 4.3	10.6 ± 4.0	*P* < 0.001*

%VC, vital capacity as percent of predicted; %FEV_1_, Forced expiratory volume in 1 second as percent of forced vital capacity.

*t test (p < 0.05).

†:BMI at diagnosis are shown. *n* = 39, 32.

‡: Respiratory function test result are shown. *n* = 30, 33.

§: Radiological findings score on CXR are shown. *n* = 50, 50.

||: Radiological findings score on CT are shown. *n* = 47, 45.

### Clinical and radiographic features of the treatment group

**Fig 3:**
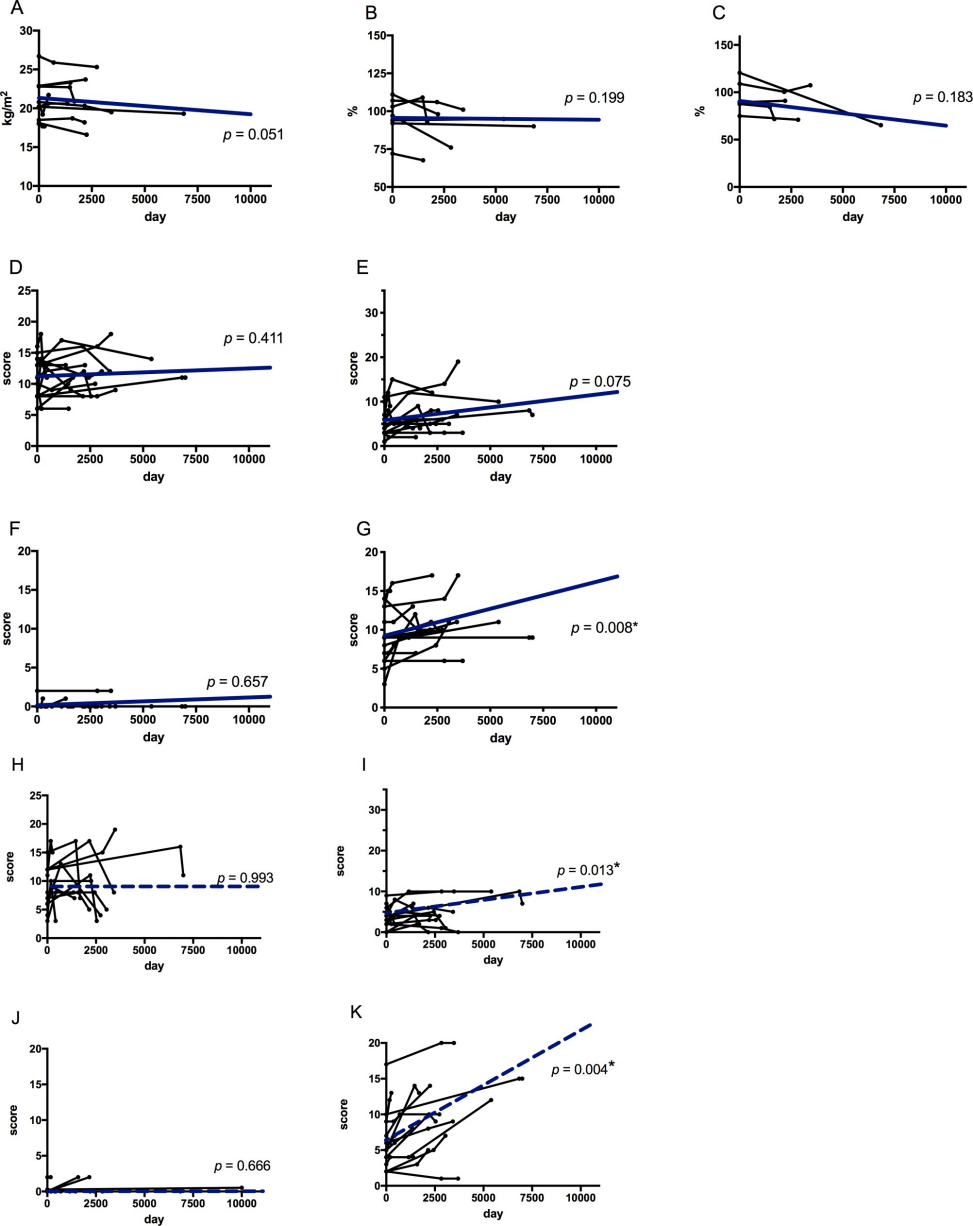
Longitudinal changes in clinical features of the treated group. The horizontal axis shows the observation period. The vertical axis shows clinical features or radiographic scores of chest X-ray (CXR) and chest computed-tomography (CT). Each bold blue line represents the average profile of the group using the mixed effect model. A: Body mass index (BMI); B: %VC (vital capacity as percent of predicted); C: %FEV_1_ (Forced expiratory volume in 1 second as percent of forced vital capacity); and radiologic scores of D: nodule (CXR), E: infiltrate (CXR), F: cavity (CXR), G: ectasis (CXR), H: nodule (CT), I: infiltrate (CT), J: cavity (CT), and K: ectasis (CT). *: Asterisks denote significant changes in the entire group through the observation period. Radiological findings score on CXR are shown. *n* = 15, 15, 15. Radiological findings score on CT are shown. *n* = 13, 13, 13.

In the treatment group (Table 3), radiographic scores of infiltrates were significantly higher at initiation of treatment than at diagnosis (CXR: infiltrates: 7.9 ± 4.4 vs 5.1 ± 2.7, *P* = 0.001; CT: infiltrates: 5.3 ± 2.6 vs 3.6 ± 2.7, *P* = 0.007, respectively at start of treatment vs at diagnosis), but those were not significantly higher at the end of follow-up (CXR: infiltrates: 6.9 ± 4.4 vs 7.9± 4.4, *P* = 0.131; CT: infiltrates: 4.1 ± 2.8 vs 5.3 ± 2.6, *P* = 0.244; respectively last visit vs at start of treatment). Overall, the scores for ectasis were significantly higher at the end of follow-up than at diagnosis (CXR: ectasis: 11.0 ± 3.3 vs 8.9 ± 3.4, *P* = 0.016, CT: ectasis: 10.3 ± 4.5 vs 9.4 ± 4.9, *P* = 0.028, respectively last visit vs at diagnosis). The BMI and pulmonary function test results did not significantly change throughout the observation period. The mixed effects model revealed that the scores for ectasis worsened significantly (CXR: ectasis: *β* = 0.000681, *P* = 0.008; CT: ectasis: *β* = 0.001529, *P* = 0.004; Fig 3), implying that treatment could slow the deterioration of clinical features and radiological findings but not completely slow progression of ectasis. Of the 10 symptomatic patients, five patients had improvement of their symptoms with treatment. Especially, haemoptysis disappeared in all cases.

**Table 3:** Longitudinal changes of clinical features in the treatment group.

	At diagnosis	At start of treatment	Last visit	*P* value		
				Before treatment	After treatment	Throughout observation
Body mass index†	21.5 ± 2.9	20.9 ± 2.7	20.2 ± 2.7	*P* = 0.626	*P* = 0.589	*P* = 0.290
%VC (%)‡	96.6 ± 12.8	95.8 ± 13.3	89.1 ± 14.7	*P* = 0.922	*P* = 0.495	*P* = 0.250
%FEV_1_ (%)‡	89.7 ± 23.1	82.5 ± 14.3	80.3 ± 24.0	*P* = 0.125	*P* = 1.000	*P* = 0.750
Radiographic scores (CXR) §						
Nodule	10.8 ± 3.1	12.1 ± 3.5	11.3 ± 2.9	*P* = 0.173	*P* = 0.266	*P* = 0.636
Infiltrate	5.1 ± 2.7	7.9 ± 4.4	6.9 ± 4.4	*P* = 0.001*	*P* = 0.131	*P* = 0.010*
Cavity	0.1 ± 0.5	0.1 ± 0.5	0.3 ± 0.6	*P* = 1.000	*P* = 0.500	*P* = 0.500
Ectasis	8.9 ± 3.4	10.3 ± 3.0	11.0 ± 3.3	*P* = 0.049*	*P* = 0.084	*P* = 0.016*
Radiographic scores (CT) ||						
Nodule	8.3 ± 3.7	12.2 ± 3.7	8.1 ± 4.7	*P* = 0.014*	*P* = 0.011*	*P* = 0.694
Infiltrate	3.6 ± 2.7	5.3 ± 2.6	4.1 ± 2.8	*P* = 0.007*	*P* = 0.244	*P* = 0.730
Cavity	0.2 ± 0.6	0.5 ± 0.9	0.4 ± 0.8	*P* = 0.593	*P* = 1.000	*P* = 0.593
Ectasis	6.5 ± 4.1	9.4 ± 4.9	10.3 ± 4.5	*P* = 0.109	*P* = 0.603	*P* = 0.028*

%VC, vital capacity as percent of predicted; %FEV_1_, Forced expiratory volume in 1 second as percent of forced vital capacity

Patients without follow-up pulmonary function testing were excluded.

*t test (p < 0.05).

†:BMI at diagnosis are shown. *n* = 13, 9, 10.

‡: Respiratory function test result are shown. *n* = 8, 5, 4.

§: Radiological findings score on CXR are shown. *n* = 15, 15, 15.

||: Radiological findings score on CT are shown. *n* = 13, 13, 13.

### Bacterial factor profiles

**Fig 4:**
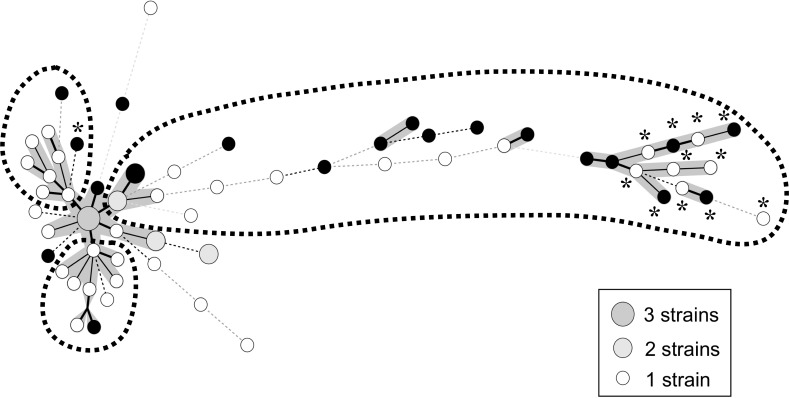
Minimum spanning tree of *Mycobacterium avium* complex isolates based on 16 loci variable-number tandem-repeat typing patterns. Each circle corresponds to a different type identified using variable-number tandem-repeat (VNTR) genotyping. Heavy lines connecting two VNTR types signify that they are single-locus variants, thin lines connect double-locus variants, and dotted lines (black) connect triple-locus variants. Black circles correspond to a strain isolated from a case for which treatment was initiated during the observation (treated group). Strains were classified into 3 general groups by dotted-line circles.

**Fig 5:**
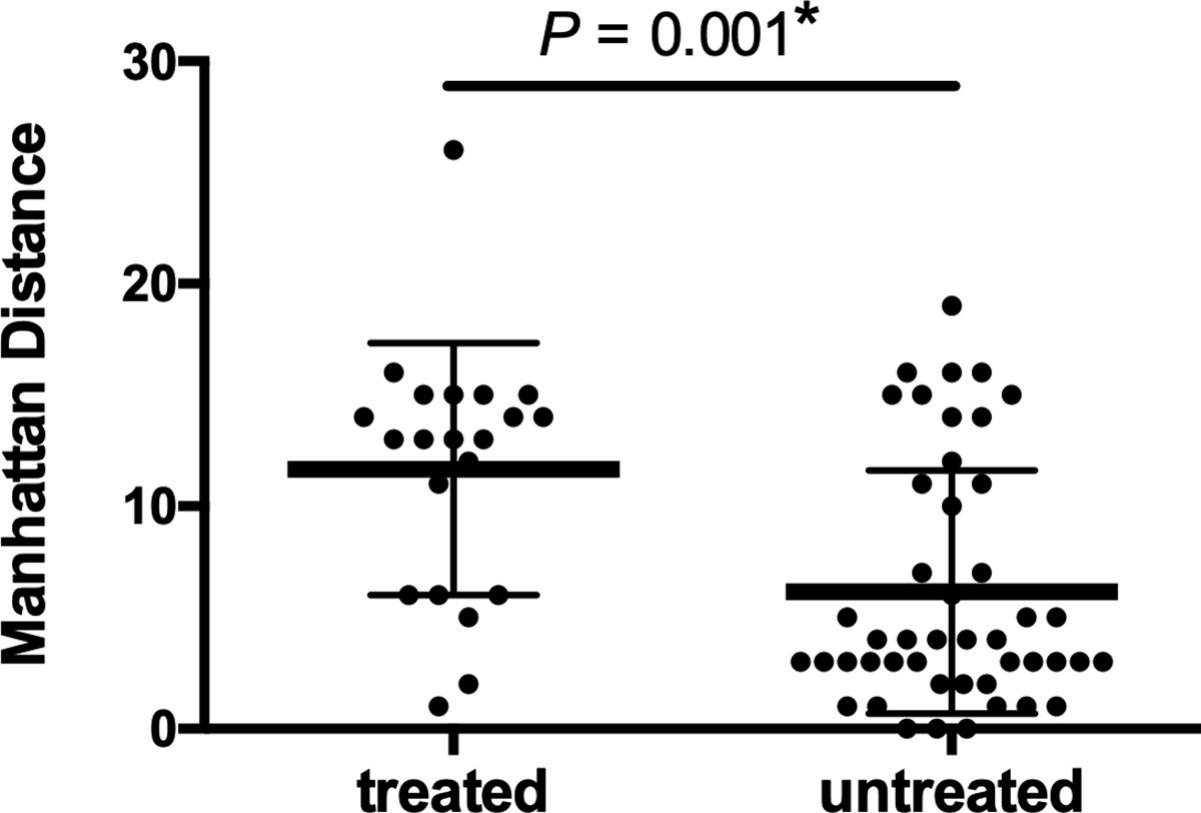
Manhattan distance from center of minimum spanning tree of *Mycobacterium avium* complex isolates. Manhattan distances were calculated based on the variable-number tandem-repeat (VNTR) pattern of center strains in the minimum spanning tree. *: Asterisks denote significant difference between the treated and the untreated groups.

Table 4 lists bacterial factor profiles. NTM species isolated from patient samples at diagnosis include *M*. *avium* (90.8%), *M*. *intracellulare* (9.2%), *Mycobacterium abscessus* (6.2%), *Mycobacterium fortuitum* (4.6%) and MAC (indistinguishable) (4.6%). There were three mixed cases with *M*. *avium* and *M*. *intracellulare*. The frequency of isolated species did not differ significantly between the observation and treatment groups. All in the treated group (15/15 cases) has shown sputum conversion. However, four cases (4/15, 26%) have developed positive cultures for MAC after the completion of treatment. The prevalence of polyclonal MAC infection at diagnosis was significantly higher in the treatment group than in the observation group (33.3% vs. 8.0%, *P* = 0.013). The two groups did not differ significantly with respect to prevalence of mixed infection, insertion sequences (IS1245, IS1311, IS*Mav6*, or IS*Mav6* in *cfp*29), or plasmid sequences (pMAH135, pLR7, pVT2, pMAH135 + pVT2, or pMAH135 + pLR7 + pVT2). The bacterial factor profile of each case is listed in S4 Table and S5 Table. Fig 4 shows the MST of *M*. *avium* isolates based on 16 VNTR loci. Strains were classified into three general groups, of which one with longest brunch was significantly associated with clinical deterioration necessitating initiation of treatment (*P* = 0.007). Manhattan distances were calculated on the basis of VNTR patterns of the strains found in the centre of the MST. These distances differed significantly between the treatment and observation groups (Fig 5, 11.7 ± 5.7 vs 6.1 ± 5.5, *P* = 0.001).

**Table 4:** Comparison of bacterial factors at diagnosis between the observation and treatment groups.

	Total n = 65 (%)	Untreated group n = 50 (%)	Treated group n = 15 (%)	*P* value
Isolated species				
*M*.*avium*	59 (90.8)	44 (88.0)	15 (100.0)	*P* = 0.060
*M*.*intracellulare*	6 (9.2)	4 (8.0)	2 (13.3)	*P* = 0.531
*M*.*fortuitum*	3 (4.6)	1 (2.0)	2 (13.3)	*P* = 0.187
*M*.*abscessus*	4 (6.2)	4 (8.0)	0 (0.0)	*P* = 0.331
MAC	3 (4.6)	3 (6.0)	0 (0.0)	*P* = 0.331
Polyclonality analyzed by VNTR				
Polyclonal + mixed infection	15 (23.1)	9 (18.0)	6 (40.0)	*P* = 0.076
Polyclonal infection	9 (13.8)	4 (8.0)	5 (33.3)	*P* = 0.013*
Mixed infection	10 (15.3)	6 (12.0)	4 (26.7)	*P* = 0.167
Insertion sequence†	(n = 55)	(n = 40)	(n = 15)	
IS 1245	49 (89.1)	36 (90.0)	13 (86.7)	*P* = 0.724
IS 1311	44 (80.0)	31 (77.5)	13 (86.7)	*P* = 0.449
IS *Mav6*	29 (52.7)	19 (47.5)	10 (66.7)	*P* = 0.205
IS *Mav6* in *Cfp29* region	13 (23.6)	11 (27.5)	2 (13.3)	*P* = 0.271
Plasmids †	(n = 53)	(n = 39)	(n = 14)	
pMAH	9 (17.0)	6 (15.3)	3 (21.4)	*P* = 0.605
pLR7	1 (1.9)	1 (2.6)	0 (0.0)	*P* = 0.545
pVT2	10 (18.9)	6 (15.4)	4 (28.6)	*P* = 0.279
pMAH + pVT2	5 (9.4)	3 (7.7)	2 (14.3)	*P* = 0.469
pMAH + pLR7 + pVT2	0 (0.0)	0 (0.0)	0 (0.0)	

VNTR, variable-number tandem-repeat.

*P < 0.05.

†: Only cases in which we could isolate and evaluate the strains of *Mycobacterium avium*.

Furthermore, we evaluated the isolated bacterial species other than NTM from all study subjects (S6 Table). There was no significant difference in detection of them between the untreated group and the treated group.

## Discussion

There is no established strategy for the management of patients with mild pMAC who do not immediately receive treatment and are managed by observation alone because the long-term natural clinical course and the effects of that treatment remain unclear. Our multicentre, retrospective study describes both the clinical course and the bacterial profiles of mild pMAC at diagnosis and without treatment. We show that polyclonal MAC infection could become a predictor of deterioration in mild pMAC, that mild pMAC slowly but substantially progresses over several years without treatment and that treatment may slow deterioration in pulmonary function, perhaps by improving reversible pulmonary lesions.

Several retrospective observational studies on the course of nodular-bronchiectatic pMAC have been published [24, 25]. Lee et al. found that almost half of 265 patients with this form of the disease required initiation of treatment during a mean follow-up of 32 months [24]. Kitada et al. report that 22.2% of 76 patients with nodular-bronchiectatic pMAC showed radiologic deterioration over a 5-yr observation period [25]. The higher percentages of patients requiring treatment in these studies indicate that these patients probably suffered from a more severe form of the disease than did the patients in our study. However, it is unclear if patients in most of the published observational studies had a history of antibiotic treatment. In contrast, and importantly, because none of the patients in our study were being treated at the start of the study, we were able to document the effects of antibiotic treatment on each outcome variable in the treatment group.

In the observation group, during the observation period, both BMI and %FEV_1_ slowly but significantly decreased, and the radiographic scores slowly but significantly increased, suggesting gradual progression of the disease regardless of the degree of symptoms at the time of diagnosis. On the other hand, the treament group showed stabilisation of BMI, pulmonary function and radiographic findings after initiation of treatment, suggesting that treatment prevented worsening of the disease. In nodular-bronchiectatic pMAC, treatment has been reported to improve cellular bronchiolitis, which is thought to be a reversible lesion [26], and pulmonary abnormalities also appear to correlate closely with pulmonary function [27]. As mild pMAC typically presents as the nodular-bronchiectatic form that includes reversible lesions, treatment would slow deterioration of pulmonary function by ameliorating reversible pulmonary abnormalities, even though bronchiectasis would remain irreversible.

We evaluated the radiological findings by using CXR and CT. There were a few discrepancies in radiographic scores between CXR and CT. Chest CT has high spatial resolution, so it can pick up and counts small lesions of nodules and ectasis, which could not be detected by CXR. Compared with CT, CXR is possible to evaluate the lesion area but not the volume of lesion. These would cause there was no prognostic predictor in CT findings at the time of diagnosis when comparing the untreated group with the treated group.

We also analysed bacterial factors affecting disease progression using samples obtained at diagnosis. Several investigators have identified various bacterial factors related to deterioration of mycobacteriosis [11, 17, 28, 29]. The prevalence of IS*Mav6* is reported to be significantly higher in the genome of pathogenic MAC strains in the Japanese and Korean populations [30, 31]. However, we found no significant difference in the prevalence of IS*Mav6* or IS*Mav6* in *cfp29* between the treatment and observation groups, and disease severity did not appear to be dependent on pathophysiology related to IS*Mav6* in *cfp29*. In addition, plasmids are gaining attention with respect to pathogenesis of pMAC. One large plasmid (pMAH135) [14] and two small plasmids (pLR7 and pVT2) [13] are known to be characteristic of clinically isolated MAC strains [12]. However, we saw no significant difference in the prevalence of these plasmids between the observation and treatment groups, and no significant association between these plasmids and VNTR patterns. Uchiya et al. have also reported that the prevalence of pMAH135 is 51.4% (18/35), which is significantly higher than that reported by us. Jucker et al. state that the prevalence of the small plasmids pVT2 and pLR7 was 58.8% (10/17) and 37.5% (6/16), respectively, which is also significantly higher than that reported by us. We think that these differences in the prevalence of plasmids may be due to regional factors.

VNTR analysis is considered to be one of the best genetic methods to discriminate among strains of both *M*. *avium* and *M*. *intracellulare* [19, 30, 32]. The results of our VNTR analysis demonstrate that the prevalence of polyclonal MAC infection in the treatment group was significantly higher than that in the observation group. The prevalence of polyclonal MAC infection in mild pMAC has been reported to be 9.0% [28] in Japan and 12.9% in Korea [33]. Interestingly, these infection rates are comparable with those of our observation group, whereas the treatment group had a considerably higher infection rate. Wallace et al. have reported that monoclonal infection was predominant in the fibrocavitary form of the disease, but that polyclonal infection was dominant in the nodular-bronchiectatic form [11]. In concurrence, our study subjects were mildly symptomatic, could be managed by observation alone for more than 6 months, and had few cavitary lesions. In contrast, patients with polyclonal infection were more likely to undergo clinical deterioration, despite early presentation with mild symptoms. Fujita et al. have reported that specific environmental exposures are associated with polyclonal MAC infection, as patients with polyclonal infections were significantly more likely to have high soil exposure, shower in a bathroom, or swim in a pool, compared with patients with monoclonal infections [28]. Thus, the nodular-bronchiectatic form may depend on particular environmental exposures, resulting in polyclonal infection and a greater likelihood of disease progression.

In the phylogenetic tree analysis of *M*. *avium*, the strains identified from the treatment group were significantly associated with one specific cluster. Similarly, some reports have suggested that disease deterioration was observed only in patients infected with strains belonging to such specific clusters [17, 29]. On the other hand, a similar study in Korea did not yield comparable results [34], and we speculate that this discrepancy could be due to differences in regional characteristics and environmental habitat of the MAC. We found that MST had a wide variety of strains, whereas the cluster of *M*. *tuberculosis* transmitted in humans is known to have a close, narrow pattern [21]. Taken together, these results could reflect the fact that the MAC is an environmental bacterium that is not transmissible among humans.

Our study has a few limitations. The major limitation to our study was its retrospective observation design. Furthermore, after our research consortium was established on July 2012, we have collected cases that we archived to follow consecutively in each hospital by July 2013. Therefore, the research period of each hospital and observation period for each patient, depending on each hospital database, were different. In addition, we had to reduce the sample size to eliminate the selection bias and exclude the cases with history of administration of treatment. However, we statistically corrected for these factors and analysed trends in the groups using a mixed effects model. Second, all of the factors such as BMI, lung functions, radiographic findings, and bacterial factors would be not independent. Further prospective research including multivariable analysis is warranted to investigate whether the results of the present study are broadly applicable.

## Conclusions

Polyclonal MAC infection is a predictive of clinical deterioration that may eventually require treatment. Mild pMAC without anti-microbial treatment showed slow but significant clinical and radiologic deterioration. Treatment can prevent deterioration due to disease but not progression of bronchiectasis.

## Supporting information

S1 Fig Criteria of inclusion and grouping. A) observation group, B) treatment group, C) exclusion; history of prescription with antimycobacterial effect, D) exclusion; short observation than six months, E) exclusion: short observation than six months without treatment.
(JPG)

S2 Fig Radiological scoring system; NICE. The lesions were defined as follows: N (nodules): Round, irregular, or branching shadows measuring up to 1 cm in diameter. I (infiltrate or consolidation): A homogeneous shadow of unspecified shape measuring 1 cm or more in diameter. C (cavity): Annular shadow at least 1 mm thick that is not a bronchus. E (ectasis): Tramline shadows and evidence of bronchial wall thickening, indicating bronchiectasis.
The separation between the upper and middle zones of each lung field was marked by a horizontal line drawn at the level of the carina. The separation between the middle and lower zones was marked by a horizontal line drawn at the level of pulmonary vein entry into the heart. The percentage of the area of each zone occupied by each of the findings (N, I, C, or E) was scored from 0 to 4 as follows: 0: 0%, 1: 1%-24%, 2: 25%-49%, 3: 50%-74%, and 4:75%-100%. A whole lung total score was calculated for each type of lesion by adding the results from 2 reviewers. The concordance rate for the scoring was evaluated with a weighted κ (Cohen, 1968). The weighted κ statistics for the radiologic evaluation were 0.965 (nodule), 0.993 (infiltrate), 1.000 (cavity), and 0.973 (ectasis). These results showed a relatively high concordance rate for scoring all 4 types of pulmonary lesions.
(JPG)

S1 Table Primers used for the amplification. The presence of plasmids in the clinical isolates was determined by amplification of the *repA* gene. Amplification of other open reading frame was tried for pMAH135 to avoid false negatives or positives.
(DOCX)

S2 Table Interobserver concordance of CXR. (a) Nodulars, (b) Infiltration, (c) Cavitary, (d) Bronchiectasis.
(DOCX)

S3 Table Interobserver concordance of CT. (a) Nodulars, (b) Infiltration, (c) Cavitary, (d) Bronchiectasis.
(DOCX)

S4 Table VNTR results of *M*. *avium*. (DOCX)

S5 Table VNTR results of *M*. *intracellulare*. (DOCX)

S6 Table Bacterial culture during follow-up (Detection rate>10%). In sputum cultures of general bacteria performed 7.0 ± 6.4 times,the report of normal flora accounted for 96.9%.
(DOCX)

## References

[1] Billinger ME, Olivier KN, Viboud C, de Oca RM, Steiner C, Holland SM, et al Nontuberculous mycobacteria-associated lung disease in hospitalized persons, United States, 1998–2005. Emerging infectious diseases. 2009;15(10):1562–9. 10.3201/eid1510.090196 19861046 PMC2866394

[2] Prevots DR, Shaw PA, Strickland D, Jackson LA, Raebel MA, Blosky MA, et al Nontuberculous mycobacterial lung disease prevalence at four integrated health care delivery systems. Am J Respir Crit Care Med. 2010;182(7):970–6. 10.1164/rccm.201002-0310OC 20538958 PMC2970866

[3] Adjemian J, Olivier KN, Seitz AE, Holland SM, Prevots DR. Prevalence of nontuberculous mycobacterial lung disease in U.S. Medicare beneficiaries. Am J Respir Crit Care Med. 2012;185(8):881–6. 10.1164/rccm.201111-2016OC 22312016 PMC3360574

[4] Morimoto K, Iwai K, Uchimura K, Okumura M, Yoshiyama T, Yoshimori K, et al A steady increase in nontuberculous mycobacteriosis mortality and estimated prevalence in Japan. Annals of the American Thoracic Society. 2014;11(1):1–8. 10.1513/AnnalsATS.201303-067OC . 24102151

[5] Al-Houqani M, Jamieson F, Mehta M, Chedore P, May K, Marras TK. Aging, COPD, and other risk factors do not explain the increased prevalence of pulmonary *Mycobacterium avium* complex in Ontario. Chest. 2012;141(1):190–7. 10.1378/chest.11-0089 . 21724552

[6] O'Brien DP, Currie BJ, Krause VL. Nontuberculous mycobacterial disease in northern Australia: a case series and review of the literature. Clinical infectious diseases: an official publication of the Infectious Diseases Society of America. 2000;31(4):958–67. 10.1086/318136 . 11049777

[7] Thomson RM; NTM working group at Queensland TB Control Centre and Queensland Mycobacterial Reference Laboratory. Changing epidemiology of pulmonary nontuberculous mycobacteria infections. Emerging infectious diseases. 2010;16(10):1576–83. 10.3201/eid1610.091201 20875283 PMC3294381

[8] Namkoong H, Kurashima A, Morimoto K, Hoshino Y, Hasegawa N, Ato M, et al Epidemiology of Pulmonary Nontuberculous Mycobacterial Disease, Japan. Emerging infectious diseases. 2016;22(6):1116–7. 10.3201/eid2206.151086 27191735 PMC4880076

[9] Aksamit TR, Philley JV, Griffith DE. Nontuberculous mycobacterial (NTM) lung disease: the top ten essentials. Respiratory medicine. 2014;108(3):417–25. 10.1016/j.rmed.2013.09.014 . 24484653

[10] Griffith DE, Aksamit T, Brown-Elliott BA, Catanzaro A, Daley C, Gordin F, et al An official ATS/IDSA statement: diagnosis, treatment, and prevention of nontuberculous mycobacterial diseases. Am J Respir Crit Care Med. 2007;175(4):367–416. 10.1164/rccm.200604-571ST . 17277290

[11] Wallace RJ Jr., Zhang Y, Brown BA, Dawson D, Murphy DT, Wilson R, et al Polyclonal *Mycobacterium avium* complex infections in patients with nodular bronchiectasis. Am J Respir Crit Care Med. 1998;158(4):1235–44. Epub 1998/10/14. 10.1164/ajrccm.158.4.9712098 . 9769287

[12] Gangadharam PR, Perumal VK, Jairam BT, Podapati NR, Taylor RB, LaBrecque JF. Virulence of *Mycobacterium avium* complex strains from acquired immune deficiency syndrome patients: relationship with characteristics of the parasite and host. Microbial pathogenesis. 1989;7(4):263–78. . 2695739 10.1016/0882-4010(89)90045-4

[13] Jucker MT, Falkinham JO 3rd. Epidemiology of infection by nontuberculous mycobacteria IX. Evidence for two DNA homology groups among small plasmids in *Mycobacterium avium*, *Mycobacterium intracellulare*, and *Mycobacterium scrofulaceum*. The American review of respiratory disease. 1990;142(4):858–62. 10.1164/ajrccm/142.4.858 . 2221593

[14] Uchiya K, Takahashi H, Nakagawa T, Yagi T, Moriyama M, Inagaki T, et al Characterization of a novel plasmid, pMAH135, from *Mycobacterium avium* subsp. *hominissuis*. PloS one. 2015;10(2):e0117797 10.1371/journal.pone.0117797 25671431 PMC4324632

[15] Hasegawa N, Nishimura T, Ohtani S, Takeshita K, Fukunaga K, Tasaka S, et al Therapeutic effects of various initial combinations of chemotherapy including clarithromycin against *Mycobacterium avium* complex pulmonary disease. Chest. 2009;136(6):1569–75. 10.1378/chest.08-2567 . 19542259

[16] Nishimura T, Hasegawa N, Fujita Y, Yano I, Ishizaka A. Serodiagnostic contributions of antibody titers against mycobacterial lipid antigens in *Mycobacterium avium* complex pulmonary disease. Clinical infectious diseases: an official publication of the Infectious Diseases Society of America. 2009;49(4):529–35. 10.1086/600888 . 19591595

[17] Kikuchi T. Disease Progression of *Mycobacterium Avium* Pulmonary Infection and The Mycobacterial Variable Number Tandem Repeat (VNTR) Typing. Kekkaku. 2013;88(1):23–7. 23513565

[18] Inagaki T, Nishimori K, Yagi T, Ichikawa K, Moriyama M, Nakagawa T, et al Comparison of a variable-number tandem-repeat (VNTR) method for typing *Mycobacterium avium* with mycobacterial interspersed repetitive-unit-VNTR and IS1245 restriction fragment length polymorphism typing. J Clin Microbiol. 2009;47(7):2156–64. Epub 2009/05/01. 10.1128/JCM.02373-08 19403768 PMC2708485

[19] Kurokawa K, Uchiya K, Yagi T, Takahashi H, Niimi M, Ichikawa K, et al Identification of Novel Variable Number Tandem Repeat (VNTR) Loci in *Mycobacterium avium* and Development of an Effective Means of VNTR Typing. Kekkaku. 2012;87(7):491–9. 22993890

[20] Ichikawa K, Yagi T, Inagaki T, Moriyama M, Nakagawa T, Uchiya K, et al Molecular typing of *Mycobacterium intracellulare* using multilocus variable-number of tandem-repeat analysis: identification of loci and analysis of clinical isolates. Microbiology. 2010;156(Pt 2):496–504. Epub 2009/10/24. 10.1099/mic.0.030684-0 . 19850613

[21] Maeda S, Wada T, Iwamoto T, Murase Y, Mitarai S, Sugawara I, et al Beijing family *Mycobacterium tuberculosis* isolated from throughout Japan: phylogeny and genetic features. The international journal of tuberculosis and lung disease: the official journal of the International Union against Tuberculosis and Lung Disease. 2010;14(9):1201–4. . 20819269

[22] Kurashima A, Morimoto K, Horibe M, Hoshino Y, Shiraishi Y, Kudoh S. A method for visual scoring of pulmonary *mycobacterium avium* complex disease: “Nice scoring system”. J Mycobac Dis. 2013;3(2):127.

[23] Hayashi M, Takayanagi N, Kanauchi T, Miyahara Y, Yanagisawa T, Sugita Y. Prognostic factors of 634 HIV-negative patients with *Mycobacterium avium* complex lung disease. Am J Respir Crit Care Med. 2012;185(5):575–83. 10.1164/rccm.201107-1203OC . 22199005

[24] Lee G, Lee KS, Moon JW, Koh WJ, Jeong BH, Jeong YJ, et al Nodular bronchiectatic *Mycobacterium avium* complex pulmonary disease. Natural course on serial computed tomographic scans. Annals of the American Thoracic Society. 2013;10(4):299–306. 10.1513/AnnalsATS.201303-062OC . 23952847

[25] Kitada S, Uenami T, Yoshimura K, Tateishi Y, Miki K, Miki M, et al Long-term radiographic outcome of nodular bronchiectatic *Mycobacterium avium* complex pulmonary disease. The international journal of tuberculosis and lung disease: the official journal of the International Union against Tuberculosis and Lung Disease. 2012;16(5):660–4. 10.5588/ijtld.11.0534 . 22410245

[26] Lee G, Kim HS, Lee KS, Koh WJ, Jeon K, Jeong BH, et al Serial CT findings of nodular bronchiectatic *Mycobacterium avium* complex pulmonary disease with antibiotic treatment. AJR Am J Roentgenol. 2013;201(4):764–72. 10.2214/AJR.12.9897 . 24059365

[27] Song JW, Koh WJ, Lee KS, Lee JY, Chung MJ, Kim TS, et al High-resolution CT findings of *Mycobacterium avium-intracellulare* complex pulmonary disease: correlation with pulmonary function test results. AJR Am J Roentgenol. 2008;191(4):1070 10.2214/AJR.07.3505 . 18806143

[28] Fujita K, Ito Y, Hirai T, Kubo T, Maekawa K, Togashi K, et al Association between polyclonal and mixed mycobacterial *Mycobacterium avium* complex infection and environmental exposure. Ann Am Thorac Soc. 2014;11(1):45–53. Epub 2013/11/21. 10.1513/AnnalsATS.201309-297OC . 24251904

[29] Nakagawa T, Takahashi H, Ichikawa K, Inagaki T, Uchiya K, Nikai T, et al Multicenter study on clinical features and genetic characteristics of *Mycobacterium avium* strains from patients in Japan with lung disease caused by *M*. *avium*. Kekkaku. 2012;87(11):687–95. Epub 2013/02/02. . 23367826

[30] Ichikawa K, Yagi T, Moriyama M, Inagaki T, Nakagawa T, Uchiya K, et al Characterization of *Mycobacterium avium* clinical isolates in Japan using subspecies-specific insertion sequences, and identification of a new insertion sequence, IS*Mav6*. J Med Microbiol. 2009;58(7):945–50. 19502362 10.1099/jmm.0.008623-0

[31] Kim SY, Jeong BH, Park HY, Jeon K, Han SJ, Shin SJ, et al Association of IS*Mav6* with the Pattern of Antibiotic Resistance in Korean *Mycobacterium avium* Clinical Isolates but No Relevance between Their Genotypes and Clinical Features. PloS one. 2016;11(2):e0148917 10.1371/journal.pone.0148917 26859598 PMC4747469

[32] Ichikawa K, Yagi T, Inagaki T, Moriyama M, Nakagawa T, Uchiya K, et al Molecular typing of *Mycobacterium intracellulare* using multilocus variable-number of tandem-repeat analysis: identification of loci and analysis of clinical isolates. Microbiology. 2010;156(2):496–504. 19850613 10.1099/mic.0.030684-0

[33] Niimi M, Uchiya K, Yagi T, Takahashi H, Kurokawa K, Ichikawa K, et al A study of genetic characteristics of *Mycobacterium avium* strains from patients with pulmonary *M*. *avium* disease in Japan and Korea. Kekkaku. 2012;87(6):461–7. . 22834098

[34] Kim SY, Lee ST, Jeong BH, Jeon K, Kim JW, Shin SJ, et al Clinical significance of mycobacterial genotyping in *Mycobacterium avium* lung disease in Korea. The international journal of tuberculosis and lung disease: the official journal of the International Union against Tuberculosis and Lung Disease. 2012;16(10):1393–9. 10.5588/ijtld.12.0147 . 23107637

