# Supplementary figures and images for: Retrospective evaluation of natural course in mild cases of Mycobacterium avium complex pulmonary disease
Source: PLoS One. 2019 Apr 25;14(4):e0216034. doi: 10.1371/journal.pone.0216034 (PMC6483267; doi:10.1371/journal.pone.0216034)

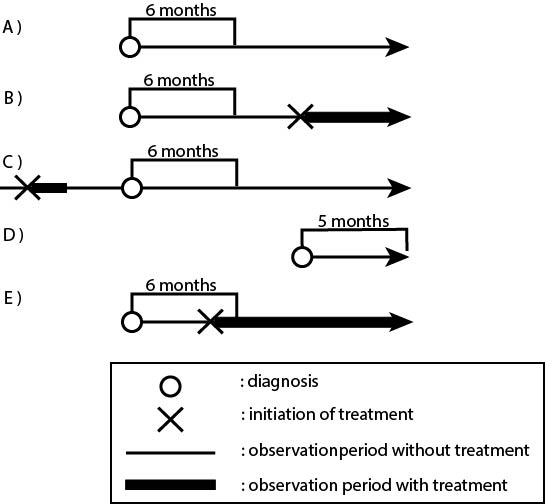

Supplement: S1 Fig — A) observation group, B) treatment group, C) exclusion; history of prescription with antimycobacterial effect, D) exclusion; short observation than six months, E) exclusion: short observation than six months without treatment. (JPG) [file pone.0216034.s001.jpg]

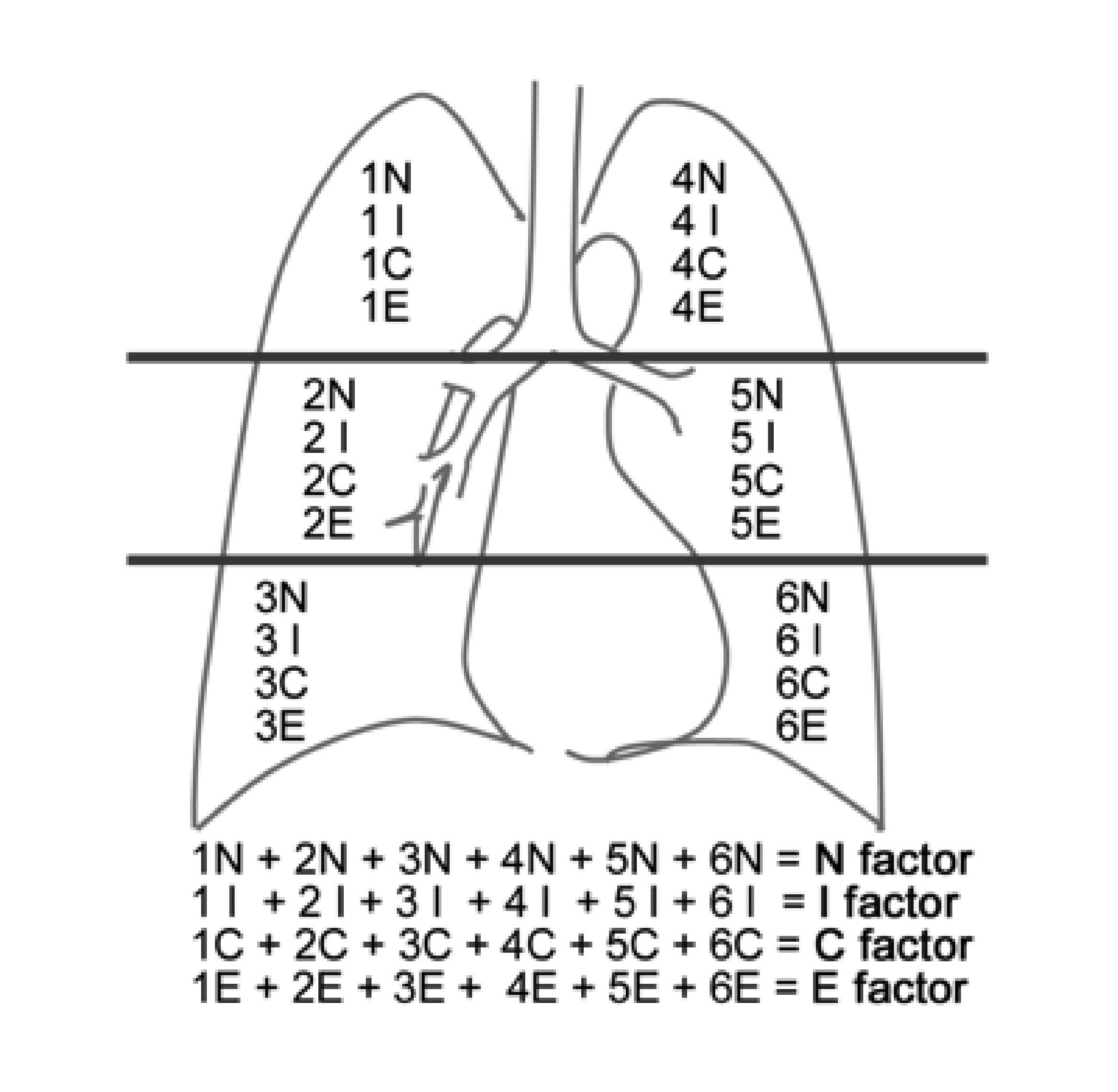

Supplement: S2 Fig — The lesions were defined as follows: N (nodules): Round, irregular, or branching shadows measuring up to 1 cm in diameter. I (infiltrate or consolidation): A homogeneous shadow of unspecified shape measuring 1 cm or more in diameter. C (cavity): Annular shadow at least 1 mm thick that is not a bronchus. E (ectasis): Tramline shadows and evidence of bronchial wall thickening, indicating bronchiectasis. The separation between the upper and middle zones of each lung field was marked by a horizontal line drawn at the level of the carina. The separation between the middle and lower zones was marked by a horizontal line drawn at the level of pulmonary vein entry into the heart. The percentage of the area of each zone occupied by each of the findings (N, I, C, or E) was scored from 0 to 4 as follows: 0: 0%, 1: 1%-24%, 2: 25%-49%, 3: 50%-74%, and 4:75%-100%. A whole lung total score was calculated for each type of lesion by adding the results from 2 reviewers. The concordance rate for the scoring was evaluated with a weighted κ (Cohen, 1968). The weighted κ statistics for the radiologic evaluation were 0.965 (nodule), 0.993 (infiltrate), 1.000 (cavity), and 0.973 (ectasis). These results showed a relatively high concordance rate for scoring all 4 types of pulmonary lesions. (JPG) [file pone.0216034.s002.jpg]
