# Supplementary material for: Retrospective evaluation of natural course in mild cases of Mycobacterium avium complex pulmonary disease
Source: PLoS One. 2019 Apr 25;14(4):e0216034. doi: 10.1371/journal.pone.0216034 (PMC6483267; doi:10.1371/journal.pone.0216034)
Supplement: S1 Table — The presence of plasmids in the clinical isolates was determined by amplification of the repA gene. Amplification of other open reading frame was tried for pMAH135 to avoid false negatives or positives. (DOCX) [file pone.0216034.s003.docx]

**S1 Table. Primers used for the amplification.**

| Target gene |  | Nucleotide sequence | Reference |
| --- | --- | --- | --- |
| pMAH135 | F: | 5’-AAA GAC GCA TTC CAC GGT-3’ | Uchiya et al., 2015 |
|  | R: | 5’-GGG GAG GTT TTA GGG AGA AA-3’ |  |
| pVT2 | F: | 5’-AAC CCT CCG GTG AGA AAG TG-3’ | Jucker and Falkinham, 1990 |
|  | R: | 5’-ACG ACG TGA GTG CGT TTG TG-3’ |  |
| pLR7 | F: | 5’-GGG TGC CAA CCA AAT CAG CC-3’ | Jucker and Falkinham, 1990 |
|  | R: | 5’-TCG CTC GGC AAG ATT CTC AG-3’ |  |

The presence of plasmids in the clinical isolates was determined by amplification of the *repA* gene. Amplification of other open reading frame was tried for pMAH135 to avoid false negatives or positives.
