# Supplementary material for: Retrospective evaluation of natural course in mild cases of Mycobacterium avium complex pulmonary disease
Source: PLoS One. 2019 Apr 25;14(4):e0216034. doi: 10.1371/journal.pone.0216034 (PMC6483267; doi:10.1371/journal.pone.0216034)
Supplement: S4 Table — (DOCX) [file pone.0216034.s006.docx]

**S4 Table. VNTR results of *M. avium***

| Patient No. | **Insertion sequence** | | | | Group | **MATR loci** | | | | | | | | | | | **HNTR loci** | | | | |  | |
| --- | --- | --- | --- | --- | --- | --- | --- | --- | --- | --- | --- | --- | --- | --- | --- | --- | --- | --- | --- | --- | --- | --- | --- |
|  | **IS *Mav6*** | **IS 1245** | **IS 1311** | ***Cfp*29 region** |  | **1** | **2** | **3** | **6** | **7** | **8** | **9** | **11** | **13** | **14** | **16** | **1** | **2** | **3** | **5** | 6 | |  |
| 1-1 | + | + | + | - | Treated | 2 | 2 | 4 | 2 | 3 | 2 | 2 | 5 | 2 | 4 | 3 | 0 | 1 | 3 | 1 | 0 | |  |
| 1-2 |  |  |  |  |  | 2 | 1 | 1 | 2 | 2 | 2 | 3 | 2 | 2 | 4 | 3 | 1 | 1 | 3 | 1 | 0 | |  |
| 2-1 | - | + | + | - | Treated | 2 | 1 | 5 | 1 | 6 | 1 | 2 | 2 | 1 | 2 | 3 | 1 | 1 | 1 | 2 | 0 | |  |
| 2-2 |  |  |  |  |  | 1 | 0 | 5 | 1 | 6 | 1 | 2 | 2 | 1 | 2 | 3 | 1 | 1 | 1 | 2 | 0 | |  |
| 3-1 | + | + | + | + | Treated | 1 | 1 | 2 | 1 | 1 | 1 | 3 | 1 | 1 | 3 | 2 | 2 | 2 | 3 | 1 | 2 | |  |
| 3-2 |  |  |  |  |  | 1 | 2 | 1 | 1 | 1 | 1 | 3 | 1 | 1 | 3 | 2 | 1 | 1 | 3 | 2 | 2 | |  |
| 4-1 | + | + | + | - | Treated | 1 | 1 | 1 | 1 | 1 | 2 | 3 | 2 | 1 | 3 | 2 | 1 | 2 | 2 | 2 | 2 | |  |
| 4-2 |  |  |  |  |  | 2 | 0 | 1 | 2 | 2 | 2 | 3 | 2 | 2 | 3 | 3 | 1 | 1 | 2 | 1 | 2 | |  |
| 5-1 | + | + | + | - | Treated | 2 | 1 | 5 | 1 | 2 | 1 | 2 | 2 | 2 | 2 | 3 | 1 | 1 | 1 | 2 | 2 | |  |
| 5-2 |  |  |  |  |  | 2 | 1 | 5 | 1 | 2 | 2 | 2 | 2 | 2 | 2 | 3 | 1 | 2 | 1 | 1 | 2 | |  |
| 6-1 | + | + | + | - | Treated | 1 | 0 | 1 | 1 | 1 | 2 | 3 | 1 | 1 | 3 | 2 | 1 | 2 | 3 | 2 | 2 | |  |
| 6-2 |  |  |  |  |  | 1 | 0 | 1 | 1 | 1 | 2 | 3 | 1 | 1 | 3 | 3 | 1 | 2 | 3 | 2 | 2 | |  |
| 7 | - | + | + | - | Treated | 1 | 1 | 5 | 1 | 6 | 2 | 2 | 2 | 2 | 2 | 3 | 0 | 1 | 1 | 1 | 0 | |  |
| 8 | + | + | + | - | Treated | 2 | 1 | 5 | 1 | 6 | 2 | 2 | 2 | 1 | 3 | 3 | 1 | 2 | 1 | 1 | 0 | |  |
| 9 | + | + | + | - | Treated | 2 | 0 | 1 | 2 | 6 | 2 | 2 | 2 | 2 | 2 | 2 | 2 | 1 | 1 | 1 | 0 | |  |
| 10 | - | + | + | - | Treated | 1 | 0 | 1 | 2 | 2 | 2 | 1 | 2 | 1 | 2 | 2 | 2 | 1 | 2 | 1 | 2 | |  |
| 11 | - | - | - | - | Treated | 2 | 0 | 1 | 2 | 2 | 2 | 2 | 2 | 2 | 2 | 3 | 2 | 1 | 3 | 1 | 0 | |  |
| 12 | - | + | + | - | Treated | 2 | 1 | 1 | 1 | 2 | 2 | 2 | 2 | 2 | 2 | 3 | 2 | 1 | 2 | 1 | 0 | |  |
| 13 | + | + | + | - | Treated | 1 | 0 | 5 | 1 | 5 | 2 | 2 | 2 | 1 | 3 | 3 | 2 | 2 | 3 | 1 | 0 | |  |
| 14 | + | + | + | - | Treated | 1 | 1 | 1 | 1 | 1 | 1 | 3 | 1 | 1 | 2 | 2 | 1 | 1 | 3 | 1 | 2 | |  |
| 16-1 | +  + | + | + | - | Untreated | 2 | 1 | 5 | 1 | 3 | 2 | 2 | 2 | 2 | 3 | 3 | 1 | 1 | 3 | 1 | 0 | |  |
| 16-2 |  | + | + | - |  | 2 | 0 | 5 | 1 | 4 | 2 | 2 | 2 | 2 | 3 | 3 | 1 | 2 | 3 | 2 | 0 | |  |
| 17-1 | + | + | + | - | Untreated | 2 | 2 | 1 | 2 | 2 | 2 | 2 | 1 | 2 | 2 | 3 | 2 | 1 | 2 | 1 | 0 | |  |
| 17-2 |  |  |  |  |  | 2 | 2 | 1 | 2 | 2 | 2 | 3 | 2 | 2 | 3 | 3 | 2 | 2 | 2 | 1 | 0 | |  |
| 18-1 | + | + | + | +/- | Untreated | 1 | 1 | 1 | 1 | 1 | 2 | 3 | 1 | 1 | 3 | 2 | 1 | 2 | 3 | 2 | 2 | |  |
| 18-2 |  |  |  |  |  | 1 | 2 | 1 | 1 | 1 | 2 | 3 | 1 | 1 | 3 | 2 | 1 | 1 | 3 | 2 | 2 | |  |
| 19-1 | - | + | + | - | Untreated | 2 | 0 | 1 | 1 | 2 | 1 | 2 | 2 | 2 | 2 | 3 | 1 | 1 | 2 | 1 | 0 | |  |
| 19-2 |  |  |  |  |  | 2 | 0 | 1 | 2 | 2 | 1 | 2 | 2 | 2 | 2 | 3 | 1 | 1 | 1 | 1 | 0 | |  |
| 20-1 | - | + | + | - | Untreated | 2 | 0 | 1 | 2 | 2 | 2 | 1 | 2 | 2 | 3 | 2 | 2 | 1 | 2 | 1 | 0 | |  |
| 20-2 |  |  |  |  |  | 2 | 0 | 1 | 2 | 2 | 2 | 1 | 2 | 2 | 3 | 2 | 2 | 2 | 2 | 1 | 0 | |  |
| 21-1 | + | + | + | + | Untreated | 2 | 0 | 1 | 1 | 2 | 2 | 2 | 2 | 2 | 2 | 3 | 2 | 1 | 2 | 1 | 0 | |  |
| 21-2 |  |  |  |  |  | 2 | 1 | 1 | 1 | 2 | 2 | 2 | 2 | 2 | 2 | 3 | 2 | 1 | 2 | 1 | 0 | |  |
| 22 | - | + | - | - | Untreated | 2 | 0 | 1 | 2 | 2 | 2 | 2 | 2 | 3 | 3 | 3 | 2 | 1 | 1 | 1 | 0 | |  |
| 23 | - | + | + | - | Untreated | 2 | 0 | 1 | 2 | 2 | 2 | 2 | 2 | 2 | 3 | 3 | 2 | 2 | 2 | 1 | 0 | |  |
| 24 | + | + | + | + | Untreated | 2 | 1 | 1 | 1 | 1 | 1 | 3 | 1 | 1 | 3 | 3 | 2 | 2 | 2 | 1 | 2 | |  |
| 25 | - | + | + | - | Untreated | 2 | 0 | 1 | 2 | 2 | 2 | 3 | 2 | 2 | 2 | 3 | 2 | 1 | 2 | 1 | 0 | |  |
| 26 | + | + | + | + | Untreated | 1 | 1 | 2 | 1 | 1 | 1 | 3 | 1 | 1 | 3 | 2 | 1 | 2 | 3 | 1 | 2 | |  |
| 27 | + | + | + | - | Untreated | 2 | 1 | 1 | 2 | 2 | 2 | 2 | 2 | 2 | 2 | 3 | 1 | 1 | 2 | 2 | 0 | |  |
| 28 | + | + | + | - | Untreated | 1 | 0 | 1 | 2 | 1 | 2 | 3 | 2 | 2 | 2 | 3 | 2 | 1 | 2 | 2 | 0 | |  |
| 29 | + | + | + | + | Untreated | 1 | 1 | 1 | 1 | 1 | 1 | 3 | 1 | 1 | 3 | 2 | 1 | 2 | 1 | 2 | 2 | |  |
| 30 | - | + | + | - | Untreated | 2 | 0 | 1 | 2 | 2 | 2 | 2 | 2 | 2 | 2 | 3 | 2 | 1 | 2 | 1 | 0 | |  |
| 31 | - | - | - | - | Untreated | 2 | 0 | 1 | 2 | 2 | 2 | 2 | 2 | 2 | 2 | 3 | 1 | 1 | 2 | 1 | 0 | |  |
| 32 | + | + | + | + | Untreated | 2 | 0 | 1 | 2 | 2 | 2 | 3 | 2 | 2 | 2 | 3 | 1 | 1 | 3 | 1 | 0 | |  |
| 33 | - | + | + | - | Untreated | 1 | 0 | 5 | 1 | 5 | 2 | 2 | 2 | 1 | 3 | 3 | 2 | 2 | 3 | 2 | 0 | |  |
| 34 | - | + | + | - | Untreated | 2 | 0 | 1 | 2 | 2 | 2 | 2 | 2 | 2 | 3 | 3 | 2 | 1 | 2 | 1 | 0 | |  |
| 35 | - | - | - | - | Untreated | 2 | 0 | 1 | 2 | 2 | 2 | 2 | 2 | 1 | 3 | 3 | 2 | 2 | 2 | 1 | 0 | |  |
| 36 | - | + | + | - | Untreated | 2 | 0 | 5 | 1 | 6 | 2 | 2 | 2 | 1 | 3 | 2 | 0 | 2 | 1 | 1 | 0 | |  |
| 37 | + | + | + | - | Untreated | 2 | 1 | 1 | 2 | 1 | 2 | 3 | 2 | 2 | 3 | 3 | 2 | 1 | 1 | 2 | 0 | |  |
| 38 | - | + | + | - | Untreated | 2 | 0 | 1 | 2 | 2 | 2 | 2 | 2 | 2 | 2 | 3 | 2 | 1 | 2 | 1 | 0 | |  |
| 39 | - | + | + | - | Untreated | 2 | 0 | 1 | 1 | 2 | 2 | 2 | 2 | 1 | 2 | 3 | 2 | 1 | 1 | 1 | 0 | |  |
| 40 | + | + | + | + | Untreated | 1 | 1 | 5 | 1 | 1 | 1 | 3 | 1 | 2 | 3 | 2 | 1 | 2 | 1 | 2 | 2 | |  |
| 41 | + | + | + | - | Untreated | 2 | 1 | 1 | 2 | 2 | 2 | 3 | 2 | 1 | 2 | 3 | 1 | 1 | 3 | 1 | 0 | |  |
| 42 | - | + | + | - | Untreated | 1 | 0 | 2 | 1 | 5 | 2 | 2 | 2 | 2 | 2 | 3 | 2 | 1 | 1 | 1 | 0 | |  |
| 43 | + | + | + | + | Untreated | 1 | 2 | 1 | 1 | 1 | 1 | 3 | 1 | 1 | 2 | 2 | 1 | 1 | 3 | 2 | 2 | |  |
| 44 | - | + | + | - | Untreated | 1 | 0 | 1 | 2 | 2 | 2 | 2 | 2 | 2 | 2 | 1 | 2 | 1 | 1 | 1 | 0 | |  |
| 45 | + | + | - | - | Untreated | 2 | 0 | 1 | 2 | 2 | 2 | 2 | 2 | 1 | 3 | 3 | 2 | 1 | 2 | 1 | 0 | |  |
| 46 | - | + | + | - | Untreated | 1 | 3 | 1 | 1 | 3 | 2 | 5 | 4 | 2 | 2 | 3 | 2 | 1 | 2 | 1 | 0 | |  |
| 47 | + | + | - | - | Untreated | 2 | 1 | 1 | 2 | 1 | 2 | 2 | 2 | 2 | 3 | 1 | 1 | 1 | 1 | 1 | 0 | |  |
| 48 | + | + | + | - | Untreated | 2 | 0 | 2 | 2 | 2 | 2 | 1 | 2 | 2 | 2 | 3 | 1 | 1 | 2 | 1 | 2 | |  |
| 49 | - | + | + | - | Untreated | 2 | 0 | 5 | 1 | 6 | 2 | 3 | 2 | 1 | 2 | 3 | 0 | 1 | 1 | 1 | 0 | |  |
| 50 | + | - | - | - | Untreated | 2 | 0 | 1 | 1 | 2 | 2 | 2 | 2 | 2 | 2 | 3 | 2 | 1 | 2 | 1 | 0 | |  |
| 51 | - | + | - | - | Untreated | 2 | 0 | 1 | 2 | 2 | 2 | 2 | 2 | 2 | 2 | 3 | 2 | 1 | 2 | 1 | 0 | |  |
| 52 | - | + | - | - | Untreated | 2 | 0 | 2 | 2 | 2 | 2 | 3 | 2 | 2 | 2 | 3 | 2 | 1 | 2 | 1 | 1 | |  |
| 53 | - | - | + | - | Untreated | 2 | 0 | 2 | 2 | 1 | 2 | 2 | 2 | 1 | 2 | 3 | 2 | 1 | 2 | 1 | 1 | |  |
| 54 | + | + | + | - | Untreated | 2 | 2 | 1 | 2 | 2 | 2 | 2 | 2 | 2 | 2 | 3 | 1 | 1 | 2 | 1 | 0 | |  |
| 104 strain |  |  |  |  | Control | 2 | 2 | 5 | 3 | 3 | 4 | 2 | 5 | 2 | 4 | 3 | 1 | 3 | 1 | 1 | 1 | |  |

* Multiple subcloning could not achieve. The isolates from No. 15 and 55 were not available for the analysis of VNTR.
