# Supplementary material for: Retrospective evaluation of natural course in mild cases of Mycobacterium avium complex pulmonary disease
Source: PLoS One. 2019 Apr 25;14(4):e0216034. doi: 10.1371/journal.pone.0216034 (PMC6483267; doi:10.1371/journal.pone.0216034)
Supplement: S5 Table — (DOCX) [file pone.0216034.s007.docx]

| **Patient No.** | **Group** | **MITR locus** | | | | | | | | | | | | | | | |  |
| --- | --- | --- | --- | --- | --- | --- | --- | --- | --- | --- | --- | --- | --- | --- | --- | --- | --- | --- |
|  |  | **1** | **2** | **3** | **4** | **5** | **6** | **7** | **8** | **9** | **10** | **11** | **12** | **13** | **14** | **15** | **16** | |
| 1 | Treated | 2 | 3 | 0 | 3 | 2 | 3 | 2 | 2 | 2 | 4 | 3 | 2 | 4 | 2 | 2 | 2 | |
| 15 | Treated | 3 | 1 | 1 | 4 | 2 | 3 | 2 | 0 | 1 | 1 | 4 | 2 | 3 | 2 | 1 | 2 | |
| 56 | Untreated | 3 | 0 | 0 | 5 | 2 | 1 | 11 | 3 | 0 | 1 | 2 | 1 | 3 | 3 | 2 | 1 | |
| 57 | Untreated | 3 | 1 | 1 | 5 | 2 | 3 | 2 | 0 | 1 | 1 | 4 | 2 | 3 | 2 | 1 | 2 | |
| 58 | Untreated | 3 | 1 | 1 | 4 | 2 | 3 | 2 | 0 | 1 | 1 | 4 | 2 | 3 | 2 | 1 | 2 | |
| ATCC13950 | Control | 2 | 3 | 2 | 3 | 2 | 3 | 3 | 2 | 2 | 4 | 2 | 2 | 3 | 2 | 2 | 2 | |

**S5 Table. VNTR results of *M. intracellulare***

* Multiple subcloning could not achieve. The isolate from No. 16 were not available for the analysis of VNTR.
