# Supplementary material for: Retrospective evaluation of natural course in mild cases of Mycobacterium avium complex pulmonary disease
Source: PLoS One. 2019 Apr 25;14(4):e0216034. doi: 10.1371/journal.pone.0216034 (PMC6483267; doi:10.1371/journal.pone.0216034)
Supplement: S6 Table — In sputum cultures of general bacteria performed 7.0 ± 6.4 times,the report of normal flora accounted for 96.9%. (DOCX) [file pone.0216034.s008.docx]

**S6 Table. Bacterial culture during follow-up (Detection rate>10%)**

| **Detected Bacteria Species** | **Detection frequency (%)** | **Untreated group**  **n = 50** | **Treated group**  **n = 15** | **Chi-Square Test (*P* value)** |  |
| --- | --- | --- | --- | --- | --- |
| *Staphylococcus aureus* | 29.2 | 14 | 5 | 0.690 | |
| *Pseudomonas aeruginosa* | 20.0 | 8 | 5 | 0.141 | |
| *Klebsiella pneumoniae* | 15.4 | 7 | 3 | 0.572 | |
| *Haemophillus parainfluenzae* | 15.4 | 7 | 3 | 0.572 | |
| *Haemophillus influenzae* | 13.8 | 5 | 4 | 0.101 | |
| *Strepptococcus pneumoniae* | 13.8 | 7 | 2 | 0.948 | |
| *Enterobacter cloacae* | 12.3 | 5 | 3 | 0.301 | |

In sputum cultures of general bacteria performed 7.0 ± 6.4 times, the report of normal flora accounted for 96.9%.
